# Economic and environmental impacts of commercial milk formula in Indonesia: estimates and comparisons using the Cost of Not Breastfeeding, Green Feeding, and Mothers’ Milk Tools

**DOI:** 10.1186/s13006-025-00732-6

**Published:** 2025-05-09

**Authors:** Nabila Nur Septiani, Andini Pramono, Tuan Thanh Nguyen, Roger Mathisen, Julie Smith

**Affiliations:** 1 IPB, IPB University, Bogor, Indonesia; 2 National Centre for Epidemiology and Population Health, Australian National University, Canberra, Australia; 3 Alive & Thrive, FHI 360 Global Nutrition, Hanoi, Vietnam; 4 College of Health Sciences, VinUniversity, Hanoi, Vietnam; 5 Crawford School of Public Policy, The Australian National University, Canberra, Australia

**Keywords:** Breastfeeding, Commercial milk formula (CMF), Cost of not breastfeeding, Green feeding, Economic cost, Greenhouse gases

## Abstract

**Background:**

Sales of commercial milk formula products (CMF) are rising rapidly. This study analysed key economic and environmental impacts CMF feeding in Indonesia, which are often overlooked in policy discussions despite their relevance.

**Methods:**

We assessed the economic and environmental impacts of CMF in Indonesia in 2020 using the Mothers’ Milk Tool (MMT), the Green Feeding Tool (GFT) and the Cost of Not Breastfeeding Tool (CONBF). We compared the estimated values from these tools with calculations based on Euromonitor data on CMF retail sales in Indonesia.

**Results:**

In 2020, according to the MMT, women in Indonesia produced around 455 million litres of breastmilk for infants aged < 6 months, which had an estimated monetary value of US$45.5 billion. The MMT and GFT shows substantial economic losses from displacement of breastfeeding in Indonesia; 62–96 million litres of breastmilk were lost in 2020 compared to the biologically feasible potential. The GFT tool calculates a carbon footprint of 215–274 million kg of CO_2_ eq. and a water footprint of 93,037 million litres. The CONBF estimates that the annual cost to families of purchasing CMF for infants aged < 24 months was US$598.6 million. By comparison, Euromonitor retail sales data suggests that in 2020, the retail value of sales of CMF products targeting the age group 0–36 months was around US$2.25 billion. Euromonitor also reports 27,200 tonnes of CMF products targeting infants < 6 months were sold in Indonesia in 2020. We calculate a carbon footprint from these sales of 299–381 million kg CO2 eq. and a water footprint of 129,064 million litres, higher than the GFT estimate.

**Conclusions:**

Breastfeeding’s economic importance to Indonesia far exceeds the retail value of CMF sales. Displacing breastfeeding carries high but largely undocumented economic and environmental costs. Losses are greater when measured as a food resource than as health costs, lost lives, or cognitive decline. Environmental impacts based on sales data are higher than those from survey data. Our findings and the discrepancies between tools reveal a critical gap in national statistics and highlight the need to recognise breast milk as an economically valuable, healthy, and sustainable national resource in Indonesia.

## Background

In recent decades, commercial milk formula (CMF) sales have escalated globally and developing countries with a high number of infants have experienced increased pressure from CMF marketing [1, 2]. The high number of infants in Indonesia makes it an important market for CMF, and Indonesia faces persistent challenges in maintaining breastfeeding rates. As in other countries, infant feeding practices in Indonesia are influenced by factors such as maternal return to work, mothers’ and infants’ health-related issues, mothers limited breastfeeding knowledge, as well as socioeconomic, healthcare, or commercial factors including industry marketing and political influence [3–12].

The health importance of breastfeeding is well known but the economic and environmental cost impacts of displacing breastfeeding with CMF products are less commonly considered. Economic costs include the financial expense to families of purchasing CMF products and additional healthcare expenses, but there are also wider economic costs which are less visible, while from the environmental perspective, there are a variety of costs of CMF production and use. Such economic and environmental factors too can influence women’s decision-making on infant and young child feeding.

Studies of the economic cost-consequences of not breastfeeding since 1996 are reviewed in a recent publication [13]. Most of these studies are for high income countries but significant economic costs have recently been documented for several countries of Southeast Asia including Indonesia [14] and globally [15]. Reliance on CMF increases household expenditures on CMF and heightens the risk of potential CMF contamination (e.g., bacterial infection) [16] which can also lead to additional healthcare costs for families. A study in the Philippines estimated families buying CMF for young children also spent a further US$143.9 million on medical care [17]. A 2018 study in Indonesia estimated the cost of not breastfeeding according to recommendation was US$118 million annually, consisting of non-medical out-of-pocket costs and healthcare system costs for treatment of diarrhoea and pneumonia/respiratory disease among young children (< 24 mo) [18].

CMF products generate waste and various harms to the environment [19–21]. High rates of breastfeeding minimise carbon and water footprints associated with CMF [22–24]. The carbon footprint from CMF for the full product life cycle (emission from production, transport, feeding equipment, and sterilisation) is estimated at 11–14 kg CO_2_ per kilogram of CMF powder [19, 25]. Feeding an infant for the whole first 6 months with CMF is estimated to generate 226–288 kg of CO_2_ [21].

Such economic and environmental costs are rarely documented comprehensively or systematically at country level. This means that despite their relevance to policy, such impacts of CMF are often overlooked in policy discussions. Three recent tools – the Mothers’ Milk Tool (MMT), the Green Feeding Tool (GFT) and the Cost of Not Breastfeeding Tool (CONBF)—now allow more complete and through analyses of direct and indirect economic costs and environmental effects for all countries where suitable data on breastfeeding practices is available. No other combination of tools can measure these consistently and spanning financial, economic as well as environmental consequences; the PROFILES program developed a computer model to assist nutrition advocacy in the 1990 s which allowed users to make financial and economic estimates of the cost of not breastfeeding and the economic value of breastfeeding for countries, using data entered by users, but did not have preloaded country data or include environmental impacts, and its development was not sustained [26]. This study aims to analyse the economic and environmental impacts of infant feeding practices in Indonesia by reporting estimates for Indonesia from the MMT, the GFT and the CONBF tools, as well as cross validating these through new calculations employing industry data on CMF retail sales in Indonesia.

Declines in breastfeeding have long been recognised as representing the loss of a nationally important food resource in Indonesia [27, 28]. These pioneering estimates of the economic value of breastfeeding and the related health system costs were published more than half a century ago [27, 28]. This made Indonesia one of the first countries in the world in which the economic value of breastfeeding was measured. Indonesia was also selected for this study as Indonesia had the highest absolute health system treatment cost of countries in Southeast Asia (Cambodia, Laos, Myanmar, Thailand, Timor-Leste, and Vietnam) [14]. As well as the economic costs of premature preventable deaths of women and children, recent studies have also highlighted the economic costs of reduced cognition in non-breastfed children, which translates into reduced educational attainment, lower future labour productivity and wages, and reduced Gross Domestic Product (GDP) growth for this very populous country which is currently targeting high-income country status [15, 29].

Methods.

### Setting

Indonesia is an upper middle-income country located in Southeast Asia. In 2023, Indonesia had a total population of 278 million, the fourth most populous country in the world. Around 4.8 million infants are born in Indonesia each year [30].

### Procedure and data

The MMT adopts the approach pioneered in Norway for estimating the national supply of human milk, and is now available online [31]; its development and methodology is documented in recent publications [32, 33]. Likewise, the GFT is available online [34] and adopts the approach of estimating the carbon footprint from CMF consumption based on survey data on infant feeding practices, as described in Smith et al. [32]. The Cost of Not Breastfeeding [35] adopts the approach of estimating the human and economic costs of not breastfeeding which is fully documented in Walters et al. [15]. Further details on these tools are below.

We used the MMT and GFT to estimate and compare human milk production and lost milk, and carbon and water footprints (using the preloaded data function) for infants aged < 6 months in Indonesia. The preloaded data on the number of infants in these tools is from the Indonesian Demographic and Health Survey (DHS) [36] and it uses the most recent breastfeeding prevalence data for Indonesia from UNICEF [37]. The CONBF also utilises this same prevalence data to estimate the economic cost of not breastfeeding.

We used retail sales data from the Euromonitor Passport Database [38] to calculate the displaced human milk, and carbon and water footprints due to CMF products sold in Indonesia, using the relevant parameters employed in the MMT and GFT, and based on assumptions from the academic literature and industry about the quantities of CMF powder and water to substitute for a litre of breastmilk.

### Using the Mothers’ Milk Tool

The MMT estimates the volume of production, ‘lost milk,’ and monetary value of breastmilk for infants aged 0–6 months for most countries (as well as for other age categories of infants and young children 0–36 months) [32]. The MMT estimates are based on the latest available survey data which was from the 2017 DHS for ‘any breastfeeding’, and recorded 4,466,000 births for 2020. The MMT’s estimation of daily breastmilk production and infant intake is conservative, with 0.7 L for infants aged 0–6 months, although for those aged 3 months, it may be closer to 0.8 L [39, 40]. The volume of breastmilk intake per child per month is the volume of breastmilk per day multiplied by 30, and the production of breastmilk per month is the number of children breastfeeding at each month of age multiplied by the volume per child per month [33]. ‘Lost milk’ is the difference between the estimated actual breastmilk production by Indonesian mothers at the surveyed prevalence of breastfeeding 0–6 months and the biological potential level if 98% of mothers were breastfeeding. To estimate the quantity of Standard Milk Formula (SMF) required to substitute for human, 1 L of SMF is taken to require 0.9 L of water and 0.129 kg of SMF powder [19, 41]. The monetary value of breastmilk is calculated from the market prices for unpasteurised donor human milk in Norway’s milk bank in 2009 at US$100 per litre [42, 43], although market prices can be much higher [32].

### Using the Green Feeding Tool

We used the GFT to estimate the carbon and water footprints from SMF use by infants aged < 6 months [23, 24]. Unlike the MMT, the GFT estimates incorporate data on ‘exclusive breastfeeding’ and ‘predominant breastfeeding’ for infants < 6 months. We used the preloaded data on exclusive and predominant breastfeeding (with plain water and non-milk liquids only) from the GFT which are based on the 2017 DHS and 4,466,000 births recorded in 2020. The preloaded prevalence in the GFT for exclusive and predominant breastfeeding is 58.2%. We used the ‘use own data’ function in GFT to input the births recorded in 2020 rather than the GFT default year of 2021, for consistency with the birth numbers in MMT, and to account for revisions in official data. We also performed calculations using exclusive breastfeeding rates of 50.7%, for comparison with the CONBF, and for 51.5%. Infants of this age who are not breastfed typically require 20–21 kg of CMF powder per 6 months of exclusive feeding of CMF [44]. In the GFT, infants who are not exclusively breastfed are assumed to have received CMF products. The GFT assumed a partially breastfed infant requires one-third of the amount of CMF or 6.7 kg and the rest is from breastfeeding. Predominantly breastfed infants are counted with the exclusively breastfed because only non-energy liquid might be added. The amount of CO_2_ equivalent greenhouse gas (GHG) generated during the lifecycle of each kilogram of CMF powder consumed is assumed as 11–14 kg [19, 25]. The water footprint generated by a kilogram of powder is taken to be 4,745 L per kilogram of CMF through the product life cycle based on previous studies [20, 21]. As in the MMT, ‘lost milk’ is the difference between the actual production of human milk and the biological potential level if 98% of mothers and infants breastfeed, but the GFT makes more precise estimates based on exclusive/predominant breastfeeding rates. As above, preparing 1 L of SMF for use requires 0.9 L of water and 0.129 kg of SMF powder.

### Using the Cost of Not Breastfeeding Tool

The CONBF takes a different approach to the economic value of breastfeeding by directly estimating the costs of not breastfeeding, using several different approaches to identifying the economic costs of breastfeeding cessation for children, mothers, health systems and society [15]. This tool relies on epidemiological and other evidence to calculate costs to families of purchasing breastmilk substitutes for infants aged 0–24 months who are not breastfed, and the associated health system costs, along with the economic costs of child and maternal mortality. The CONBF calculation uses a 2017 prevalence of exclusive breastfeeding among infants < 6 months in Indonesia of 50.7%, continued breastfeeding of 54.6%, and early initiation of breastfeeding of 58.2% [37].

### Using Euromonitor data

For this study, we follow the terminology used by Euromonitor International in its *Baby Food Market Reports.* SMF and special baby milk formula (SBMF) are CMF products sold at retail in Indonesia for infants (< 6 months), with follow-up milk formula (FUF), and growing-up milk (GUM) targeting older infants and young children.

To cross-validate estimates from the three tools, we firstly used retail sales data from the Euromonitor Passport Database to manually quantify the volume of SMF sold in Indonesia in 2020. This enabled comparisons between MMT estimates of lost breastmilk based on ‘any breastfeeding’ rates, and SMF retail sales data which offer insights into potential MMT underestimation from limitations arising from using survey data on any breastfeeding prevalence.

Secondly, carbon and water footprints estimated from the GFT were compared with estimates calculated using data from the Euromonitor database. These allowed us to more accurately estimate carbon and water footprints, again by addressing potential underestimation due to the GFT using survey data on exclusive breastfeeding prevalence. This also provided insights into actual SMF retail sales volumes in Indonesia, allowing comparisons with GFT estimates of lost breastmilk based on ‘exclusive or predominant’ breastfeeding rates.

Finally, we validated CONBF data on costs to families of purchasing breastmilk substitutes for infants and young children aged < 24 months, which are imputed from survey data of household expenditures, by comparisons with Euromonitor data on retail sales of CMF products marketed for children aged < 36 months.

## Results

### Retail sales data on SMF and SBMF in Indonesia

**Fig. 1:**
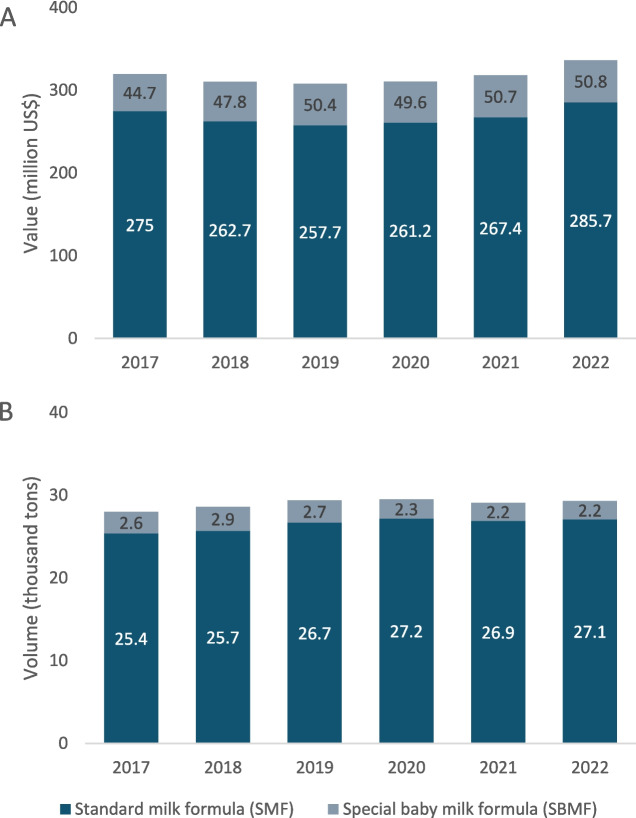
Sales of standard milk formula (SMF), special baby milk formula (SBMF) in Indonesia from 2017–2022 in millions of US$ (**A**) or thousands of tonnes (**B**)

The Euromonitor Passport Database provides the value and volume of CMF retail sales in Indonesia for each year from 2017–2022. The highest value of SMF and SBMF sales were in 2022 at US$285.7 million and US$50.8 million. The highest volume of SMF sales was in 2020, reaching 27,200 tonnes that year, while for SBMF the peak was in 2018 at 2,900 tonnes (Fig. 1).

Comparison of the MMT estimates with manual calculations using the Euromonitor Passport database.

In 2020, the estimated total breastmilk production for infants aged < 6 months in Indonesia was 454.63 million litres based on ‘any breastfeeding,’ with a biologically feasible production of 517.06 million litres if 98% of women were enabled to breastfeed optimally (Table 1). The monetary value of this breastmilk was estimated by MMT at US$45.5 billion, significantly higher than the US$261.2 million recorded in the value of sales of SMF, according to the Euromonitor Passport database.

**Table 1:** Comparison of breastmilk production estimates using Mothers’ Milk Tool (MMT) and retail sales of standard milk formula (SMF) based on Euromonitor data for 2020

Variables	Estimates using MMT	Estimates based on Euromonitor data
Volume of breastmilk produced (million litres)	454.63	-
Biologically feasible potential production of breastmilk (million litres)	517.06	-
Lost breastmilk (million litres)	62.42	210.85^a^
Volume of SMF powder sales (million kg)	-	27.2
Value of SMF sales (US$ million)		261.2

Data source: Volume and value of SMF sales from Euromonitor Passport Database [38]; Mothers’ Milk Tool [31]

^a^The calculation of lost breastmilk implied from Euromonitor data on commercial milk formula (CMF) sales uses the following equation: 1 L/0.129 kg) × 27.2 million kg = 210.85 million litres

The MMT estimates that 62.42 million litres of breastmilk were lost in 2020, representing about 12% of the potential total if most mothers could breastfeed exclusively for six months. The lost human milk estimate implies a loss of economic value to Indonesia of around US$6.2 billion in 2020. In contrast, the sale of 27.2 million kg of SMF powder corresponds to an estimated displacement of 92.4 million litres of breastmilk, nearly three times the lost breastmilk estimates indicated by the MMT.

### Comparison of footprints from the GFT and the Euromonitor Passport database

In 2020, the GFT estimated the lost breastmilk in Indonesia at 96.1 million litres. The carbon footprint from SMF consumed in the country ranged between 215.3 and 274.1 million kg of CO_2_ equivalent emissions, and the total water footprint for SMF estimated at 93,037 million litres (Table 2). According to the Euromonitor Passport Database (2022), 27.2 million kg of SMF was sold in Indonesia, resulting in a carbon footprint of 299.20–380.80 million kg of CO_2_ equivalent and a water footprint of 129,064 million litres. When comparing the two estimates, the carbon footprint from the Euromonitor data was approximately 38.95% higher than the GFT estimate, while the water footprint was about 38.72% higher. These differences highlight that the carbon and water footprint estimates based on the SMF sales data were significantly higher than those derived from the GFT.

**Table 2:** Comparison of carbon and water footprint estimates using Green Feeding Tool (GFT) and retail sales of standard milk formula (SMF) based on Euromonitor data for 2020

Variables	Estimates using GFT	Estimates based on Euromonitor data
Volume of breastmilk produced (million litres)	454.63	-
Lost breastmilk (million litres)	96.1	210.85^a^
Volume of SMF powder sales (million kg)	-	27.2
Value of SMF sales (US$ million)		261.2
Carbon footprint (CO_2_ eq., million kg):		
Lower estimation	215.3	299.2
Upper estimation	274.1	380.8
Water footprint (million litres)	93,037	129,064

Data source: Volume and value of SMF sales from Euromonitor Passport Database [38]; Green Feeding Tool [34]

^a^The calculation of lost breastmilk implied from Euromonitor data on commercial milk formula (CMF) sales uses the following equation: 1 L/0.129 kg) × 27.2 million kg = 210.85 million litres

### Estimating the economic cost of not breastfeeding from the CONBF

The CONBF illustrates a range of economic costs of not breastfeeding incurred by Indonesia (Table 3). The annual expense incurred by families for CMF in Indonesia is estimated by the CONBF tool at almost US$600 million a year to feed children CMF up to 24 months. By comparison, Euromonitor data suggests the value of SMF and SBMF retail sales targeting infants < 6 months was US$310.8 million in 2020, and CMF product sales targeting children 6–36 months were around $US2.25 billion. Hence the Euromonitor total averages an annual US$900 million for each year of the 0–36 months age category, compared to the CONBF annual average of US$300 million.

**Table 3:** Comparison of annual costs of commercial milk formula (CMF) in Indonesia; CONBF tool and Euromonitor retail sales, 2020

Variables	CONBF	Euromonitor	
	All CMF products	SMF and SBMF	FUF and GUM
CMF purchases and sales (US$ million)	598.6	310.8	2,253
Health system costs (US$ million)	60.1	-	-
Economic costs of morbidity (US$ million)	4,300.0	-	-
Economic costs of mortality (US$ million)	611.5	-	-

Data source: Cost of Not Breastfeeding Tool (CONBF) [35] and sales of standard milk formula (SMF), special baby milk formula (SBMF), follow-up milk formula (FUF), growing-up milk (GUM) from Euromonitor Passport Data [38]

The CNBF tool also estimates that health system costs of not breastfeeding are around US$ 60.1 million a year in Indonesia for maternal (breast-and ovarian cancer, type 2 diabetes) and child (diarrhoea, acute respiratory infections/pneumonia, obesity) conditions. The economic costs of morbidity and lost lives are much larger for infants who are not breastfed in the first 6 months of life totalling nearly US$5 billion a year.

## Discussion

### Novel analysis of Indonesia’s economic and environmental impacts of CMF

CMF retail sales, including SMF marketed for infants aged < 6 months in Indonesia result in significant economic losses and environmental impacts which remain largely unrecognised. This is the first country case study integrating analyses of the economic and environmental impacts of premature cessation of breastfeeding. We use three innovative tools, MMT, CONBF, and GFT. We also compare and integrate the tool results using preloaded data from the official survey on feeding practices with results from using data on CMF sales for Indonesia from the Euromonitor Passport database. Our estimates of human milk production, lost breastmilk, and carbon and water footprints illustrate the foregone economic value and the harmful environmental impact of consumption of CMF in Indonesia. We also show the substantial expense to families, to the health system, and to the Indonesian economy and productivity of preventable deaths arising from the expansion of the CMF market in Indonesia.

### Economic impact of SMF consumption displacing breastfeeding

The MMT estimates show that the estimated monetary value of breastmilk lost in Indonesia due to displacement by SMF is around US$6.2 billion in 2020. Despite the perceived economic importance of the SMF industry [45], the value of SMF sales is only US$261 million. This is much lower than the economic value of breastmilk produced by women for this < 6-month infant age group, estimated at US$45.5 billion annually by the MMT. However, the MMT underestimates lost breastmilk production in Indonesia. This is because for internal consistency with available UNICEF datasets for all age groups 0–36 months, the MMT uses ‘any breastfeeding’ data to calculate both milk production and lost milk. Using ‘any breastfeeding’ as benchmark means it underestimates the lost milk for the exclusive breastfeeding period < 6 months.

The GFT complements the MMT by providing more refined estimates of lost breastmilk for the < 6 months age group using the more specific UNICEF [37] datasets available for that age group, which identify both exclusive and predominant breastfeeding. Using these more precise measures of exclusive and predominant breastfeeding, the GFT estimated lost breastmilk at 96.1 million litres in 2020, which is considerably higher than the estimate using ‘any breastfeeding’ data in the MMT (62.42 million litres). Slight differences in the preloaded birth data in the MMT and GFT do not substantially affect the comparisons. Nevertheless, there are slight differences in the GFT between the preloaded data and manual calculations using the own data function for the three age groups < 6 months, caused by the lack of suitable data availability.

A unique aspect of this study is integrating data on SMF sales volumes from Euromonitor with results on lost milk estimated by the MMT and GFT. SMF sales volumes from Euromonitor were converted to liquid equivalents of breastmilk displaced and indicate a greater displacement of breastfeeding by SMF in 2020 (210.85 million litres) than the MMT or GFT suggests.

These findings reinforce that the MMT estimate of economic losses due to lost breastmilk significantly underestimates the true scale of the economic impact of rising SMF consumption [46, 47], and the GFT provides a more accurate measure of lost milk for the 0–6 months age group.

Furthermore, the CONBF tool indicates CMF purchases for children aged 0–24 months amounting to around US$598 million annually in Indonesia for all CMF product categories, while sales data from Euromonitor show higher annual levels even after accounting for the broader age category (0–36 months) in the latter dataset. Both estimates confirm the large scale of CMF product use in Indonesia, even though different coverage and methodologies for their data collection are likely to explain some of the discrepancy in their estimates. Specifically, the CONBF estimate is for feeding CMF products from birth till age 24 months, and the average price paid by households for CMF products was conservatively imputed based on the lowest price, economy brand product [15]. The Euromonitor data is for products sold for ages up to 36 months and is based on actual prices of CMF products sold in Indonesia. Hence the higher result from Euromonitor sales compared to the CONBF is not unexpected.

### Environmental impacts of SMF consumption

As well as having economic implications, SMF consumption can also impact the environment, including GHG emissions from the use of SMF. Reducing the production and consumption of animal-based food, such as meat and dairy products, is important to reducing environmental emissions and health impact [48]. A study from six Asia Pacific countries excluding Indonesia in 2012 estimated the GHG emissions from SMF at over 18 billion miles of car travel [49]. Our study confirms that Indonesia, as a growing market for SMF, is a significant source of GHF emissions in Southeast Asia, further emphasizing the global environmental implications of SMF consumption [22, 49].

Our findings on environmental impacts of SMF using the GFT also suggest that the carbon and water footprint of SMF is much higher when measured using Euromonitor sales data on SMF (299–381 million kg of CO_2_ eq. GHG emissions) than when estimated based on survey data on infant feeding practices (215–274 million kg of CO_2_ eq. GHG emissions) such as used in the GFT.

Overall, our comparisons of results from calculations using Euromonitor data on SMF retail sales suggest that the MMT and the GFT underestimate both the economic and environmental impacts of SMF marketed for infants aged < 6 months in Indonesia, with an unexplained discrepancy between results from the tools based on breastfeeding prevalence and SMF sales data of about 100 million litres of lost milk.

There may be several reasons for this. It is possible that SMF is used for older infants, as well as those aged < 6 months. SMF sold in Indonesia may be informally exported to other countries without this being fully reflected in Euromonitor data. The amount of SMF fed to infants may be overestimated based on retail sales volumes because of high rates of waste due to households complying with advice to throw away unused formula after each bottle feed or because parents may over-dilute it to reduce the expense.

However, these are unlikely to account for the large difference between MMT and GFT estimates and those implied by data for SMF sales [38]. Although SBMF is excluded from our calculations, its inclusion would widen the discrepancy. The size of the difference suggests that the discrepancy arises because the MMT and GFT tools rely on DHS survey data collections that do not fully capture the extent of the use of CMF from birth. The MMT is based on any breastfeeding rather than exclusive breastfeeding, while both the MMT and the GFT use survey data on breastfeeding in the previous 24 h, rather than since birth. As a result, both the tools and particularly the MMT can be understood to underestimate the extent of CMF use and the associated economic and environmental impacts calculated from national data on infant and young child feeding practices.

### Implications for policy and monitoring

Overall, we suggest that the economic value of breastfeeding, as well as the economic losses and associated environmental impacts of SMF, are likely to be much greater than previously indicated by the MMT and GFT tools. Integrating the GFT component to the lost breastmilk estimation in the MMT and economic costs in the CONBF can widen and strengthen advocacy for breastfeeding, including actions to achieve the Global Nutrition Targets for breastfeeding and subsequently reduce the consumption of CMF.

This has important implications for monitoring infant and young child feeding practices in Indonesia and globally, as it points to concerns about whether current survey data accurately reflects reality. The measurement of exclusive breastfeeding of infants aged < 6 months is conventionally based on surveys collecting feeding data from mothers based on maternal recall of feeding practice for the last 24 h. Indonesia relies on this methodology in the DHS [38]. However, it has been shown that measurement of infant feeding practice for the last 24 h will result in a considerable overestimation of the prevalence of exclusive breastfeeding from birth and potentially lead to disastrous policy complacency about rates of exclusive breastfeeding [50]. Aarts found difference in the exclusive breastfeeding rate was over 40 percentage points at both 2 and 4 months of age, (92% versus 51% at 2 months and 73% versus 30% at 4 months) and 9 percentage points at 6 months (11% versus 1.8%) [50]. This order of magnitude is consistent with our findings in this study.

Fifty years after the large economic value of breastfeeding by Indonesian women was first drawn to attention, the continued lack of recognition of the large economic value and cost savings of breastfeeding in official statistics and policymaking and the lack of awareness of the substantial societal, economic and environmental costs of CMF products may contribute to continued unregulated and exploitative CMF marketing and inadequate policy support to enable more women and children to breastfeed.

Importantly, the increase in SMF sold in Indonesia is measured as an increase in its GDP, but the much larger loss of breastmilk production associated with not breastfeeding was not measured and so the large economic loss of breastmilk is invisible to policymakers [27, 28, 51]. Our estimates show that the monetary value of breastmilk produced by Indonesian women is far higher than the retail value of SMF sold in the same year. This indicates the potential for misplaced priorities in policymaking [52, 53] due to the invisibility of women’s important production of breastmilk in Indonesian economic statistics.

Our findings have implications for public policy including for monitoring of infant feeding practices.

Breastmilk is a nationally important food resource for infants and children and should be measured and monitored to inform policy or program measures to protect, promote, and support breastfeeding [54]. Not including breastmilk production in food production and economic statistics can lower its importance in the eyes of policymakers, with an unsupportive social environment for breastfeeding adversely impacting women’s ability to supply their milk to the nation’s children. Lack of recognition of the importance of breastfeeding is reflected, for example, in inadequate maternity care support for breastfeeding and weak regulation of SMF marketing including through the health system [46, 55–57], and insufficient fiscal priority to invest in maternity protections for working women [58]. This also means that the crucial capital represented by women’s traditional breastfeeding knowledge and skills is depreciated into the future [33].

### Strengths and limitations

A key contribution of this study is that it is the first to estimate both the loss of breastmilk and the GHG emissions resulting from SMF consumption for a country, in this case, Indonesia. Our results are crucial for raising awareness among parliamentarians, government officials, employers, mothers, breastfeeding counsellors, and healthcare workers about the importance of protecting, promoting, and supporting breastfeeding in Indonesia.

No previous studies have examined the environmental implications of SMF use in Indonesia, although the MMT builds on pathbreaking studies of the economic value of breastfeeding in Indonesia in the 1980 s [27]. Additionally, using the Indonesian case study, we tested for the first time the validity of using DHS survey data on infant feeding as a proxy for measuring the carbon and water footprints of SMF using the GFT.

Our use of multiple analytical tools (Mothers Milk, Green Feeding and CONB) provides a comprehensive picture of the financial, economic, and environmental impacts of CMF at the country level, and through the triangulation of different data and tool methods, our study generates a more robust and insightful impact analysis.

However, there are some limitations to our estimates, which point to even greater SMF consumption in Indonesia and call for future investigations. Euromonitor data excludes distributions of SMF products through health facilities and institutions, which can be considerable. Additionally, suitable data was lacking for more precise estimates for the < 6-month infant age group in the MMT design but could be incorporated in future enhancements of the tool if more detailed datasets become available. The GFT design is similarly limited by the availability of suitable data on infant and young child diets beyond the 6-month age group.

Future research could use the same methods to calculate economic and environmental impacts of CMF for other countries. However, improved data collection on infant and young child feeding practices is essential for enhancing the accuracy of estimates. This is particularly true for high-income countries where data collections on infant and young child feeding practices are poor [59], yet the economic and environmental impacts are potentially very high due to low breastfeeding rates and high production and use of CMF products.

The high volume of SMF sales including compared to the lower estimates of purchases also raises the question of whether SMF may be being used for older infants or young children. We were not able to consider undocumented amounts of CMF that have been imported or exported from Indonesia. The discrepancy might signal overfeeding, stock losses, or high plate waste. With available data, this research was unable to capture issues related to infant and young children’s actual dietary intake or exports of SMF.

The case study method limits generalisability of our results; however, future research can consider using similar methods for other country cases especially in Southeast Asian countries. Our approach of comparing retail sales data on CMF with results from the tools using DHS or similar survey data on infant and young child feeding could be replicated to provide estimates for other countries and regions, offering scientific evidence for policy advocacy and dialogue.

Furthermore, these tools can be used in maternity care settings as one of main distribution channels of CMF. However, data in facility level is lacking and should be collected nationally.

## Conclusions

Breastfeeding is a substantial and valuable component of the national food system in Indonesia. The US$45 billion monetary value of breastmilk provided by breastfeeding mothers for infants far exceeds the US$261 million of SMF retail sales during 2020. The ongoing displacement of breastfeeding has high but largely undocumented economic and environmental costs. Economic losses are greater when measured as a food resource rather than health costs, lost lives, or cognition losses.

Our results expose a critical gap in national statistics, underscoring the need to recognize breastmilk as a valuable, healthy, and sustainable national resource essential for food security in Indonesia. Governments should consider how food production statistics and food balance sheets can be improved and developed to address the information gap on availability of this high-quality food for infants and young children.

The environmental impacts of current feeding practices are equally troubling. The carbon and water footprints associated with CMF are significantly higher than those for breastfeeding. This study highlights that the environmental impacts of CMF sales are likely underestimated, even if made visible by tools such as the GFT. The discrepancy between GFT estimation and SMF sales data underscores a significant gap in understanding the true environmental costs of CMF and point to a need for more accurate assessments and better data integration. There is an urgent need for detailed study of why DHS and Euromonitor data do not closely align, including the extent to which DHS survey methods underestimate the prevalence of exclusive breastfeeding for the whole of the recommended period from birth to six months.

The GFT complements the MMT, and both are essential to recognising the importance and productivity of women in breastfeeding for human health and environmental sustainability. The CONBF is essential for helping policymakers and advocates understand the direct health and financial costs of not breastfeeding and the potential economic benefits that could result from government and development partners’ investments in improving effective breastfeeding promotion, protection, and support strategies.

Breastfeeding in Indonesia is likely to be significantly undermined by the undervaluation of breastmilk's economic and environmental importance, as demonstrated by these tools.

Addressing these issues requires improving data accuracy, policy development, and regulatory practices. Incorporating detailed data on the economic value of breastmilk and the environmental impacts of CMF into policy frameworks is essential for promoting breastfeeding and mitigating CMF’s adverse effects. Enhanced regulation of CMF marketing and increased breastfeeding support in maternity care settings are crucial for improving public health and environmental sustainability. By recognising and addressing the true costs of SMF consumption, stakeholders can develop more effective and equitable strategies to support breastfeeding, protect maternal and infant health, and foster a more sustainable and productive society.

A further study recommendation includes applying these tools in maternity care settings, as the health services sector is known to have high GHG emissions, and supplementation can determine the success of the breastfeeding journey.

## Data Availability

No datasets were generated or analysed during the current study.

## Abbreviations

CMF: Commercial Milk Formula
CONBF: Cost of Not Breastfeeding Tool
DHS: Demographic and Health Survey
FUF: Follow-up Milk Formula
GFT: Green Feeding Tool
GHG: Greenhouse Gas
GUM: Growing-up Milk
MMT: Mother’s Milk Tool
SBMF: Special Baby Milk Formula
SMF: Standard Milk Formula
